# Evaluation and differentiation of the *Betulaceae* birch bark species and their bioactive triterpene content using analytical FT-vibrational spectroscopy and GC-MS

**DOI:** 10.1186/1752-153X-6-67

**Published:** 2012-07-18

**Authors:** Simona Cîntă-Pînzaru, Cristina A Dehelean, Codruta Soica, Monica Culea, Florin Borcan

**Affiliations:** 1 Molecular Spectroscopy Dept., Babeş-Bolyai University, 1st M. Kogălniceanu Str., Cluj-Napoca, 400084, Romania; 2 Faculty of Pharmacy, Victor Babeş University of Medicine and Pharmacy, 2nd Eftimie Murgu Sq., Timişoara, 300041, Romania; 3 Biomedical Physics Department, Babeş-Bolyai University, 1st M. Kogălniceanu Str., Cluj-Napoca, 400084, Romania

**Keywords:** Natural extracts, Betulin, Betulinic acid, Lupeol, FT-Raman, FT-IR

## Abstract

**Background:**

Aiming to obtain the highest triterpene content in the extraction products, nine bark samples from the forest abundant flora of Apuseni Mountains, Romania were Raman spectroscopically evaluated. Three different natural extracts from *Betula pendula Roth* birch bark have been obtained and characterized using Fourier transform vibrational spectra.

**Results:**

This study shows that principal components of the birch tree extract can be rapidly recognized and differentiated based on their vibrational fingerprint band shape and intensity. The vibrational spectroscopy results are supported by the GC-MS data. Based on IR and Raman analysis, one can conclude that all the extracts, independent on the solvent(s) used, revealed dominant betulin species, followed by lupeol.

**Conclusions:**

Since Raman measurements could also be performed on fresh plant material, we demonstrated the possibility to apply the present results for the prediction of the highest triterpene content in bark species, for the selection of harvesting time or individual genotypes directly in the field, with appropriate portable Raman equipment.

## Background

Because of its wide beneficial pharmacological activities, the bark of birch tree has been the subject of respect since ancient times, as well as the more recent subject of exhaustive research and natural extracts industry [1]. Betulin can be actually considered the first active principle isolated from a vegetal source [2]. The potential of plant origin products has been reevaluated for the use in still incurable diseases such as cancer and AIDS [1]. Many previously published reports have revealed the occurrence of lupane-based triterpenes across a multitude of plant specie [3].

Currently it is generally accepted that the outer bark of birch tree is rich in pentacyclic triterpene compounds such as betulinic acid (BA, 3β-hydroxy-20(19)-lupaen-28-oic acid), betulin (B, lup-20(29)-ene-3*β*,28-diol), lupeol (L, Lup-20(29)-en-3-ol) and other minor components, such as oleanolic acid, ursolic acid and betulinic aldehyde [4]. The pentacyclic triterpenes are known to have a wide-range of pharmaceutical activities, among them possessing anti-virus, anti-inflammatory, anticancer and other properties [5]. Betulin reaches the highest percentage within the composition of triterpenes from the birch bark [6] and exhibits significant therapeutic activity, acting as antitumor, antiviral and antiseptic agent. [4] Betulinic acid, the oxidation product of betulin, reveals important anti-HIV-1 activity and specific cytotoxic activity against different tumor cell lines [3]. Betulin and betulinic acid have been involved in chemical modulation, leading to highly active derivatives, some of them comparable to clinically used drugs [3,7].

Summarizing previous data findings, the betulin content is dominant, whereas the betulinic acid content is much lower [8]. However, plant species from different geographical locations might reveal a variable composition of the extraction product, which is essential to be accurately characterized for biological use, including the content in BA and B.

During the last years, especially infrared spectroscopy (IR) has been introduced as a very efficient and non-destructive analytical tool for the reliable identification of triterpenes resulted from various extraction methods. [9-12] Our previous study [9] combined for the first time complementary FT-IR and FT-Raman techniques for characterization of the birch three leaves, buds and bark. However, the limits of NIR spectroscopy technique require complementary analysis methods for identification of the expected compounds. So far, a great number of NIR calibrations have been perfected in order to predict in a simultaneous manner the amount of volatile and non-volatile substances in different plants; as opposed to NIR measurements, the fundamental absorption bands noticed in the mid-infrared (MIR) range can offer concrete information regarding functional groups of the individual species [12].

Raman spectroscopy is currently recognized as a flexible, accurate and high sensitive technique, that is currently widely applied in a plenty of domains, as recently excellent reviewed the current [13] and former editor of the topic journal [14-16].

Since the *Betulaceae* species are largely represented in the European flora, they represent a valuable resource of potentially pharmaceutical products and therefore must be investigated in a nondestructive, rapid and sensitive manner. Thus, the identification of birch bark material in close relation to the geographical location is crucial in ensuring the quality and efficacy of the raw sample before it is converted to the final product. Natural product companies are continuously seeking a faster and inexpensive validation method considering that the traditional wet chemistry analysis does not offer these advantages.

In this work, we employed FT-Raman and FT-IR spectroscopy in conjunction with GC-MS to characterize the raw bark and natural extract products obtained from the *Betula pendula Roth* species, abundant in the Aninei Mountains, Romania. Additionally, nine bark samples harvested from a birch forest in Apuseni Mountains, Romania, have been Raman investigated, in order to evaluate the spectral response from different specimens and to use this material as reference point for extracts. Further aim of these investigations was to evaluate the potential of FT-vibrational spectroscopy to directly identify the main active compounds from birch bark natural extract products.

## Results and discussion

**Figure 1:**
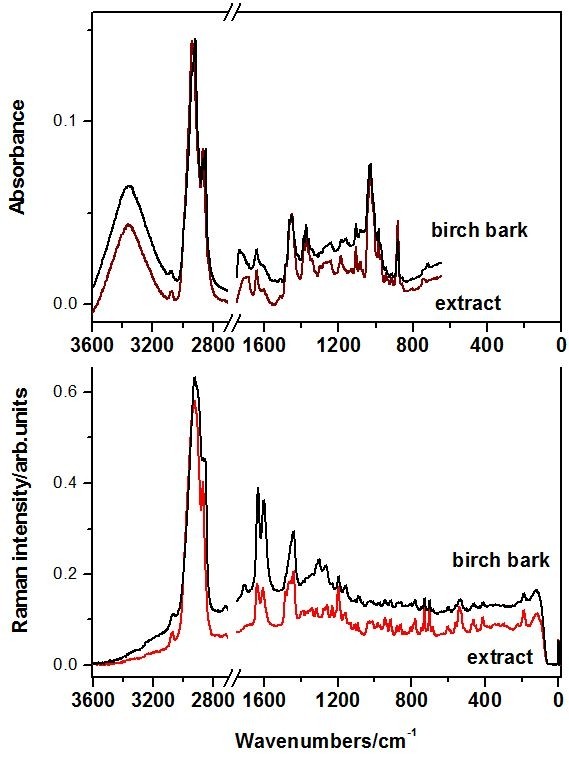
**ATR-FT-IR and FT-Raman spectra of the raw birch bark that exhibits the highest I**_**1633**_**/I**_**1601**_**Raman ratio and the extract product BC1.**

FT-vibrational spectra of the raw birch bark from Aninei Mountains and one of the extract products, BC1, are shown in the Figure 1. At a first glance, the spectral pattern is rather similar, exhibiting same main band positions and relative intensities both in the IR and Raman. Small differences are observed in the 1788–1633 cm^-1^ IR absorbance as well as in the 1400–1250 cm^-1^ Raman range. As the extraction product is consistent with the pentacyclic triterpenes structure as confirmed by the GC-MS, the vibrational FT-IR and Raman spectra directly revealed the triterpenes specific content of the raw bark.

**Figure 2:**
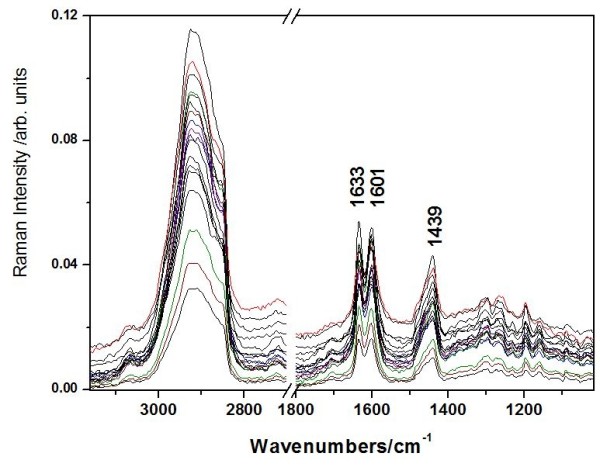
**FT-Raman spectra collected from two distinct bark points from each of nine birch trees from spontaneous forest flora area in the Apuseni Mountains, Romania.** The relative intensity of the bands at 1633 cm^-1^ representative for the triterpenes and 1601 cm^-1^ assigned to other species than triterpenes, I_1633_/I _1601_ varies from 0.9 to 1.1 in the nine bark samples.

In order to evaluate the possible variation of triterpene content from one tree to another, and from one area to another, nine different birch located in our area were subject of Raman investigation. Figure 2 presents the FT-Raman spectra collected from two distinct bark points from each of nine birch trees from spontaneous forest flora area in the Apuseni Mountains, Romania. These raw materials were used to evaluate in detail with vibrational spectroscopy the birch bark composition as a reference point for extracts and to prove the applicability of this technique for such an application.

The spectral shape is rather similar with a specific major difference. The relative intensity ratio of the band at 1633 cm^-1^ representative for the triterpenes [6,9,17,18] and the band at 1601 cm^-1^ assigned to other species than triterpenes, I_1633_/I_1601_ varies from 0.9 to 1.1 in the nine samples. This variation depends on a lot of factors like the age of the tree, the sun exposure, the soil and others. The origin of the band at 1601 cm^-1^ is uncertain, since many organic compounds exhibit such band. According to our previous vibrational assignments of betulinic acid [17] and betulin [6], this band is absent from the triterpene spectra. Therefore, the band at 1601 cm^-1^ could suggest other wood content associated to the impurities resulted from extraction protocol (Figure 1).

### IR and Raman spectra of the extraction products

**Figure 3:**
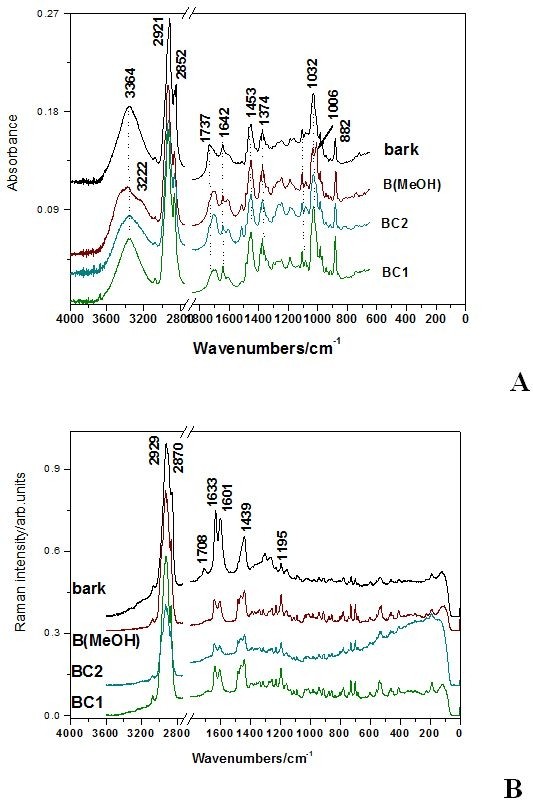
The ATR-FT-IR (A) and FT-Raman spectra (B) of the three extract products compared to those of the raw birch bark.

The ATR-FT-IR and FT-Raman spectra of the obtained extract products are displayed in the Figure 3 A and B, respectively, in comparison with the spectrum of the raw birch bark that exhibits the highest I _1633_/I _1601_ Raman ratio.

### IR Analysis

The FT-IR spectral pattern of all the three extracts is rather similar, exhibiting the same main band positions and relative intensities. Small differences are observed in the 1788–1633 cm^-1^ IR absorbance range. IR absorbance of the three extracts revealed rather similar molecular composition, based on the main characteristic IR absorption of the triterpene. Similarities with the spectrum of the pure, unprocessed bark are remarkable. Exception in the spectral feature is observed for the band at 1735 cm^-1^ attributable to the ν(C = O) mode, that is prominent in the bark spectrum.

MeOH solvent from extraction protocol also introduces differences in the spectrum of the corresponding extract (specific bands at 882 and 3222 cm^-1^, Figure 3A.).

**Figure 4:**
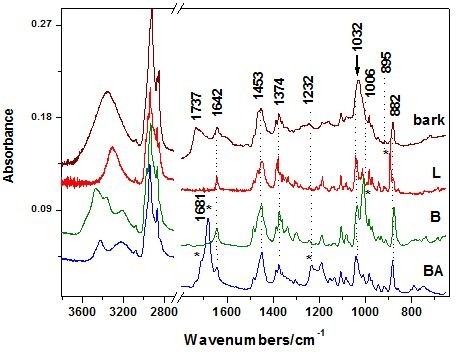
**The ATR-FT-IR spectra of the reference compounds, betulin, betulinic acid and lupeol in comparison with the absorbance of raw bark.** The * symbol denotes the specific band of each reference compound that could be used for triterpenes differentiation.

ATR-FT-IR spectra of the three extract products have been compared to those reported from isolated compounds extracted from the *Betula utilis* and *Hyptis suaveolens* from Hymalaya [11] or Betulaceae cortex, *Betula pendula Roth*[10] from Central Europe, reported so far. The ATR-FT-IR spectra of the reference compounds, betulin, betulinic acid and lupeol are displayed in the Figure 4, in comparison with the IR absorbance of raw bark. The * symbol denotes the specific bands of each reference compound that could be used for triterpenes differentiation.

The IR band positions observed in our spectra and their assignments are summarized in the Table 1, in comparison with the IR main bands of betulin, betulinic acid and lupeol, recorded in the 4000–650 cm^-1^ range. The band positions marked in bold are suitable for triterpenes differentiation and correspond to those with the symbol * in Figure 4.

**Table 1:** **The observed IR bands (cm**^**-1**^**) of the extract compounds of Betulaceae species, and the pure references species betulin (B), lupeol (L) and betulinic acid (BA) together with their assignments based on our previous DFT calculations**[3]**of betulin**

**IR**	**IR**	**IR**	**IR**	**IR**	**IR**	**Assignments**
***Betula utilis*extract from ref.**[11]	**Isolated triterpenes from*Betula Pendula Roth,*ref.**[10]	**Extract BC1 from*Betula Pendula Roth***	**Betulin (B)**	**Betulinic acid (BA)**	**Lupeol (L)**	
*3433 (B)*	-	-	3471vs	3431	-	*ν* (OH)
-	-	-	3465 sh	-	-	*ν* (OH))
-	3384 (BA)	3362 s	3362 s	-	3309 s	*ν* (OH)
	3348 (B)					
	3308 (L)					
-	-	-	3212 m	3225	-	*ν* (OH)
-	-	2940 vs	2968 vs	2943 vs	2982 s	*ν*_as_(CH_3_) + *ν*_as_(CH_2_) + *ν*_as_(CH)
					2957 s	
					2942 vs	
-	-	-	2929 vs	-	2928 sh	*ν*_as_(CH_2_) + *ν*(CH_2_) + *ν*(CH)
-	2870 (BA)	2867 s	2866 vs	2878 vs	2874 vs	+ *ν*(CH_2_+ *ν*(CH_2_)
	2868 (B)				2845 sh	
	2872(L)					
***1725 (B) 1672 (BA)***	1686, BA	1709-1684, broad	1735 w, 1708 sh	**1716** sh, **1681 vs**	-	***ν*(C = O)**
1648 (B)	1638)(B)	1642	1642 m	1642 m	1642 m	δ(CH_2_) + ν((C = C) + δ(C-C-H)
	
	1630 (L)					
*1600 (B) 1560 (B)*	-	1601	-	-	-	-
-	1468 (L) 1432 (BA, B)	1484 m 1452 s	1485 m 1450 s	1451	1453 m	δ(CH_3_) + δ(CH_2_)
-	1362 (O-H, BA); 1380 (L) 1388 (B)	1373 m	1373 m	1377 m	1374 m	δ(CH_3_) + δ(CH_2_)
1230 (B) 1240(BA)	-	-	-	**1232 m**	-	δ(OH) + τ(CH_2_) + δ(CH)
-	1156 (BA)	1190 m	1190 m	1190 m	1190 m	ν(C-C) + δ(OH) + τ(CH_2_) + δ(CH)
1028 (BA)	920 (L)	1028 s	1032 ms	1043 m	1046 m	ν(C-O) from CH_2_-OH, + δ(CH) + ρ(CH_3_, CH_2_)
-	-	1006 sh	**1006 vs**	-	*-*	*ν*(C-O) + *δ*(CH) + *ρ*(CH_3_-CH_2_)
		984 m				
881 (B)	920 (L)	881 ms	875 ms	885 ms	**895 s**	*ω* (H-C-H) umbrella alkene

Abbreviations: v-very, s- strong, m-medium, sh-shoulder, ν -stretching, δ -bending, τ -torsion, ρ-rocking; B-betulin, BA-betulinic acid, L-lupeol.

Analyzing the IR spectra of the isolated compounds from Betulaceae extracts with dry column chromatography, a recent study [10] concluded that triterpene substances betulin, betulinic acid and lupeol, isolated from external birch bark (*Betula Pendula Roth*) give identical, characteristic signals and absorbance as reference standards. Those spectra have been recorded using KBr pellet technique. However, the reference standard IR spectra reported cannot differentiate between betulin and lupeol. Furthermore, other reference [11] claims that authentic betulin exhibits distinctive bands at 3430, 1716, 1641, 1600, 1581,1291, 881 cm^-1^, whereas betulinic acid at 3508, 1710, 1690, 1641, 1600, 1580, 1290 cm^-1^, as summarized in the Table 1 for comparison.

Taking a closer inspection to the Figure 4 it is noticed that the band at 1600 cm^-1^ is absent in the reference spectra. Its origin is associated with other organic compounds than triterpenes, as mentioned above. Moreover, the band shape in the range 1032–1006 cm^-1^ of the bark reflects the triterpene contribution, since betulin is responsible for the contribution at 1006 cm^-1^ whereas betulinic acid and lupeol exhibit contribution at 1032 cm^-1^ (Figure 4). Betulinic acid exhibits a strong characteristic IR band at 1681 that allow fast differentiation among triterpene species. However, this fingerprint is not distinct observable in the bark spectrum, suggesting that its contribution content is minor. The high wavenumbers range of the bark spectrum is less suitable for triterpenes differentiation, since the bark IR contribution is an envelope of the tree specific spectral features (Figure 4). These results suggest that IR spectroscopy allows differentiate betulin, betulinic acid and lupeol, in spite of their high structural similarity. IR only could provide information for triterpene differentiation and quantitative estimation in the 1032–1006 cm^-1^ absorbance range of the raw bark or the extract product. Keeping this in mind, we are able to direct evaluate and compare the three extract products concerning the triterpenes content/impurity ratio. We calculated the relative intensity ratio of the bands I_1032_/I_1601_ whose IR spectra are shown in the Figure 3A. The values of 3.32, 1.7 and 1.47 for the BC1, BC2 and B(MeOH) extracts respectively, suggest that BC1 extract presents the highest purity. Furthermore, evaluation of the relative betulin content could be achieved using the betulin IR fingerprint band at 1006 cm^-1^. Consequently, the corresponding ratios of 2.29, 1.519 and 1.52 respectively, suggests that BC1 extract exhibits the betulin highest content, whereas the others revealed similar lower content, independent on the solvent(s) used. The vegetal tissues contain complex mixtures of substances known to possess synergistic activities. [19,20] Structure-activity relationship studies [7,21] revealed that C-28 carboxylic acid of betulinic acid was very important for its cytotoxicity. A recent study for the simultaneous extraction and determination of betulin and betulinic acid in white birch bark using RP-HPLC has been reported. [22] The extraction was tested using different solvents, leading to the conclusion that 95% ethanol in water is a good extraction solvent which allowed extraction of triterpenes at a highest content. Separation was achieved on a reversed phase C_18_ column with acetonitrile/water 86:14 (v/v). Detection was accomplished with UV detection at *λ*=210 nm. The bioactive triterpenes in white birch bark were simultaneously determined, significant variations in the content of betulin and betulinic acid in white birch bark from different China locations being observed [22].

### FT-Raman analysis of the extraction products

FT-Raman spectra of the three extracts shown in the Figure 3B exhibit similar spectral pattern concerning the band positions and relative intensities. Major difference is noted in the Raman background, generally associated with fluorescence impurities. The highest signal-to-noise ratio has been obtained from the BC1 sample followed by B(MeOH). The natural birch bark spectrum exhibits characteristic Raman bands of the extract products, assigned to the pentacyclic triterpene, besides other components (extra bands at 1708 and 1301 cm^-1^).

**Figure 5:**
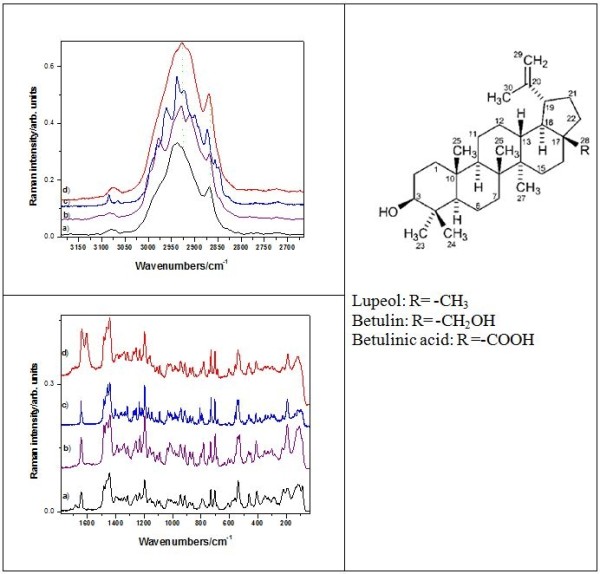
High (top) and low (bottom) wavenumbers spectral range of the FT-Raman spectra of the betulinic acid (a), betulin (b), lupeol (c) and BC1 extract (d). Excitation: 350 mW, 1064 nm. The molecular structure of the three lupan skeleton compounds is shown in the right.

**Figure 6:**
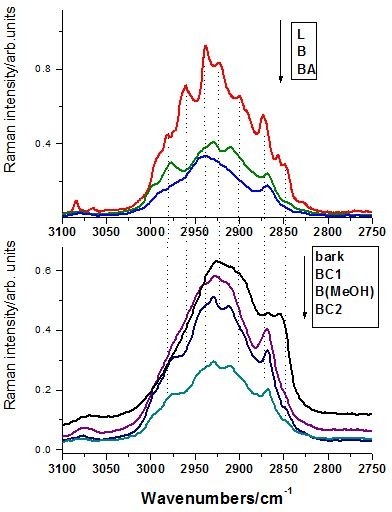
**Bottom: high wavenumber range of the FT-Raman spectra of the BC1, B(MeOH), BC2 extracts and bark (bottom), compared with the reference spectra of betulin (B), lupeol (L) and betulinic acid (BA), respectively, (top).** The arrow indicates the display order. Excitation: 350 mW, 1064 nm.

**Figure 7:**
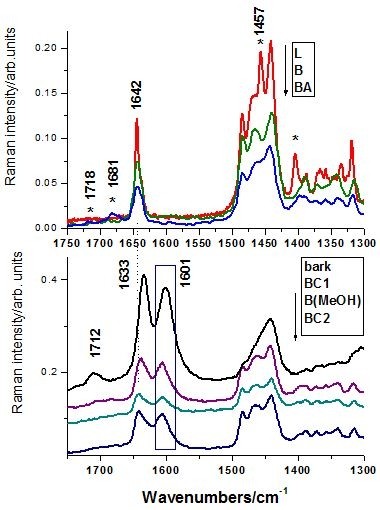
**Low wavenumber range of the FT-Raman spectra of the BC1, B(MeOH) and BC2 extracts and bark (bottom), compared with the reference spectra of betulin (B), lupeol (L) and betulinic acid (BA), respectively (top).** The arrow indicates the display order and the rectangle illustrate the band not attributable to triterpene species. Excitation: 350 mW, 1064 nm.

In order to characterize the Raman behavior of the extracts, the reference Raman spectra of the betulinic acid betulin and lupeol have been loaded (Figure 5). The three lupan skeleton compounds are similar, the difference being the –COOH, –CH_2_–OH and –CH_3_ functional groups, respectively. These differences are reflected in the Raman spectra by the presence of the specific distinct bands, as shown in detail in the Figure 6 and 7 for the high and low wavenumbers range, respectively. The Raman spectra of the three extracts, BC1, B(MeOH), and BC2 respectively are also shown in the same bottom figure (Figures 6 and 7), for eye guidance and a better comparison.

Since the best Raman signal/noise ratio has been recorded in the case of BC1 extract, its FT-Raman spectrum was displayed in comparison with the pure betulin, lupeol and betulinic acid species spectra. Taking a closer look at the high wavenumber region (Figure 6), one can observe that the BC1 Raman bands follow the envelope of the three reference spectra, with the maximum centered on betulin representative band and shoulders characteristic for clearly resolved bands of lupeol. Because of the band shape complexity and overlap, this Raman spectral range does not allow clear triterpene species differentiation. In the low wavenumbers range (Figure 7), betulinic acid exhibits specific weak Raman bands (IR strong) at 1718 and 1681 cm^-1^ (marked with * symbol), whereas lupeol showed strong Raman fingerprint at 1457 and 1408 cm^-1^ assigned to the methyl deformation modes (Table 1). These fingerprint bands are less discernable individually in the triterpene mixture due to the weak intensity in the case of BA, which is less representative in the overall extract content. Raman spectrum of the BC1 extract contains all the characteristic bands of the pure betulin, located at 2979 (shoulder), 2929, 2909, 2870, 1642, 1486, 1465, 1441, 1194, 780, 732, 702 cm^-1^. These bands are present in all the extraction products or in the raw bark spectra (Figures 6 and 7). Additionally, a distinct strong band located at 1601 cm^-1^, is constantly observed (Figure 7, rectangle pointed out), also showed above in IR spectrum. It must be pointed out that the C=C Raman mode of the three triterpene is located at 1642 cm^-1^, whereas the raw bark showed this broaden band centered at 1633 cm^-1^. A possible explanation would be the presence of other aromatic compound(s), which would be also responsible for the “impurity” band at 1601 cm^-1^. In conclusion, the betulin spectral Raman signature is dominant in the Raman spectra of the extracts, suggesting its content dominance, whereas betulinic acid and lupeol are not directly detectable due to their weak overall Raman contribution within overlapped betulin bands. Raman spectroscopy could provide quantitative information concerning the triterpene content in the final extract products by analyzing the relative intensity ration of the 1642 cm^-1^ (C=C mode) and 1601 cm^-1^ band, assigned to other organic residual compounds from the extraction protocol. The latter band provenience is the raw bark and not the solvents used as confirmed by the Raman analysis of different bark samples.

A wide range of Raman investigation of distinctive substances in several medicinal and aromatic plants have been reported [16-18,23] and the key Raman bands were discussed by comparison to the corresponding pure standard components. Raman spectra obtained directly from vegetal tissue by using laser excitation in the visible range are often completely covered by the huge fluorescence background and consequently, the inherent weak Raman scattered signal is not observable. To overcome such aspects, NIR laser sources such as laser diode (785 nm) or Nd:YAG laser emitting at 1064 nm are employed, or other Raman techniques, such as Resonance Raman or SERS [24] for the plants investigation with visible laser lines.

### GC/MS analysis of the extracts

**Figure 8:**
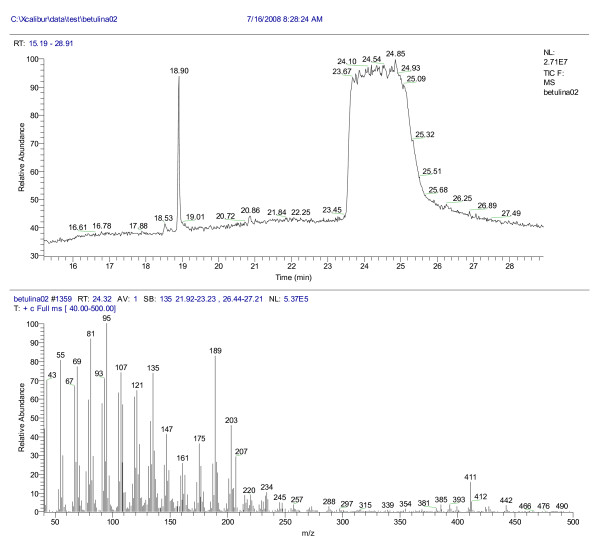
**The chromatogram and mass spectrum of betulin (ethanol/methanol, elution time 23.7 min)**. Tcol:50 °C(1 min)20 °C/min l;a 310 °C(15 min); M=442.

**Figure 9:**
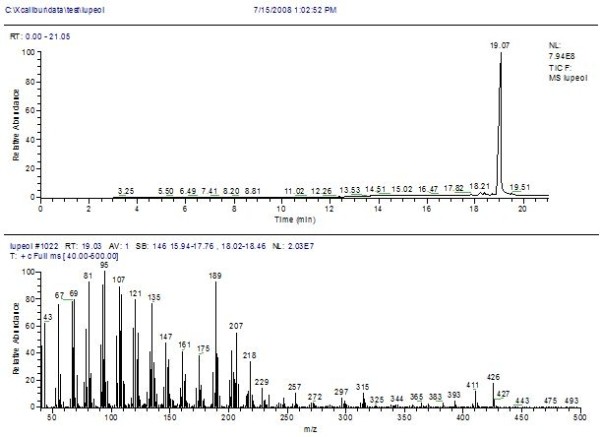
**The chromatogram and mass spectrum of lupeol, (ethanol/methanol, elution time 19.07 min).** Tcol:50 °C(1 min)20 °C/min l;a 310 °C(15 min); M=426.

Mass spectra of betulin (M=442, elution time 23.7 min) and lupeol (M=426, elution time 19.07 min) are displayed in the Figures 8 and 9, respectively.

The separation chromatogram of the BC1 extract is also attached in the Figure 9. As expected, two major compounds, betulin (58.8%) and lupeol (41%) were dominant in the BC1 extract, as indicated by the mass spectra, whereas the other two extracts, BC2 and B(MeOH) respectively, exhibited less triterpene content [25] (22.8% and 23.7% betulin, respectively), in agreement with the predicted vibrational data. The NIST library of spectra was used for reference.

The main observations regarding this study include the possibility of Raman techniques application for in detail elucidation of important antitumor compounds like pentacyclic triterpenes. Another important point is the possibility of comparison between important techniques like vibrational spectroscopy, gas chromatography on elucidation of extracts purity and their composition easy applicable even in case of pentacyclic triterpenes with lupan skeleton. The elucidation for extraction procedure is an indicator about the most acceptable extraction route (e.g. solvent type, quantity, type of procedure). As we expected the extracts contain lupan skeleton triterpenes but their percentage in the extract differ because of the procedure and solvents. Continuous extraction seems to be the most quantitative for these compounds.

## Methods

### Plant material

The plant material, outer bark of birch tree, *Betula pendula Roth*, (*Betulaceae*) was harvested from the Aninei Mountains (Banat region, South Weastern of Romania) in October 2010. Voucher samples were deposited in the Herbarium of the Department of Pharmaceutical Botany of the Faculty of Pharmacy, University of Medicine and Pharmacy, Timisoara, voucher BB1265/10. Only the outer part of the bark (the cork) that spontaneously separates from the stem in autumn was used. The cork was air-dried at room temperature, broken in small pieces and ground into powder by a mill. All solvents were reagent grade. The raw sample of birch bark was collected from 4.85 g outer bark dried at room temperature. The extracts were prepared from the outer bark by Soxhlet extraction in methanol as solvent. [8,24] BC1 sample was obtained from 5.01 g bark in 250 ml methanol; the extraction has been performed for 2 hours. It was selected after comparation with another two types of extractions, using a Soxhlet (Gerhardt) continuos extraction: BC2 sample resulted from 4.93 g birch bark and 250 ml cloroform/diclormetan/metanol 1:1:1 solvents mixture for 2 hours, continuous extraction and B(MeOH) sample from 4.90 g dried sample with 250 ml methanol using a ultrasonication bath (Falc). According to the literature [1,9,10,20,26] using a mixture of solvents could result in an increased extraction products amount but we proved that the continous methanolic extract assure a good extraction yield for triterpene content. [8] In order to establish the end of extraction, the depletion of vegetal material was used. The dried forms were obtained by using a Büchi rotary evaporator device. Additional bark samples from nine birch three (vouchers BB1291-1299/10) were collected in October 2010 for Raman measurements.

Isolation and GC-MS analysis of triterpenes has been achieved from the purified samples solved in methanol/dichloromethane.

### Apparatus

A Trace DSQ Thermo Finnigan quadruple mass spectrometer coupled with a Trace GC was employed for GC/MS analysis. A Rtx-5MS capillary column, 30 m length x 0.25 mm, 0.25 μm film thickness was used in a temperature program from 50 °C, kept 1 minute, then with 20 °C/minute to 310 °C, kept for 15 min.

FT-IR and FT-Raman spectra have been recorded using an Equinox 55 Bruker spectrometer with an integrated FRA 106 S Raman module. A Nd:YAG laser operating at 1064 nm line was employed for the excitation of the powdered sample extract or the raw birch bark. The laser power was set to 350 mW. An attenuated total reflectance (ATR) MIRacle module with ZnSe contact crystal has been coupled to the Equinox 55 FTIR Bruker spectrometer for the recording of the ATR-FT-IR absorbance spectra in the 4000–650 cm^-1^ range. The spectral resolution was 2 cm^-1^ and 40 scans were accumulated for each powder sample. The spectral data have been processed using Origin 8.0 software.

## Conclusions

We employed vibrational FT-IR and FT-Raman spectroscopy as rapid, sensitive and efficient techniques for qualitative and semi-quantitative analysis of the pentacyclic triterpene based natural products as highly effective anticancer agents obtained from the *Betula pendula Roth* birch bark. Nine bark samples from the abundant forest flora of Apuseni Mountains, Romania were Raman evaluated concerning their triterpenes content. Additionally, three different extraction conditions have been applied for one birch bark specimen aiming to obtain the highest triterpene content in the extraction product. Methanol solvent and longer extraction time revealed the product with highest triterpene content as revealed by the vibrational analysis. The conclusions were supported by the GC-MS analysis that revealed two major compounds, betulin (59%) and lupeol (41%) in the highest purity extract.

All the three extracts presents similar main IR/Raman spectral fingerprint associated to the pure triterpenes, where betulin bands were dominant. Betulinic acid is less observable as distinct compound in the extraction product, on one hand, due to its low concentration and on the other hand due to the weak Raman intensity characteristic for the –COOH group at 1681 and 1718 cm^-1^.

Based on IR and Raman analyses, one can conclude that all the extracts, independent on the solvent(s) used, revealed dominant betulin species, followed by lupeol. Raman spectroscopy could provide semi-quantitative information concerning the triterpene content in the final extract products by analyzing the relative intensity ration of the 1642 cm^-1^ (C=C mode) and 1601 cm^-1^ band, assigned to other organic residual compounds from the bark. Since Raman measurements could also be performed on fresh plant material, we demonstrated the possibility to apply the present results for the prediction of the highest triterpene content in bark species, for optimal harvest time or for the selection of individual genotypes directly in the field, with appropriate portable Raman equipment.

## Competing interests

The authors declared that they have no competing interests.

## Authors’ contribution

SCP carried out the analysis design and natural products screening studies. CAD conceived the study and participated in its design. CS participated in the analysis of the products and helped to draft the manuscript. MC and FB carried out the GC-MS analysis and participated in the interpretation of results. All authors read and approved the final manuscript.
